# Five novel *RB1* gene mutations and genotype–phenotype correlations in Chinese children with retinoblastoma

**DOI:** 10.1007/s10792-022-02341-2

**Published:** 2022-08-12

**Authors:** Luting Li, Haibo Li, Jing Zhang, Hairun Gan, Ruihong Liu, Xinyan Hu, Pengfei Pang, Bing Li

**Affiliations:** 1 Department of Interventional Medicine, The Fifth Affiliated Hospital of Sun Yat-Sen University, Zhuhai, 519000 People’s Republic of China; 2 Guangdong Provincial Key Laboratory of Biomedical Imaging and Guangdong Provincial Engineering Research Center of Molecular Imaging, The Fifth Affiliated Hospital of Sun Yat-Sen University, Zhuhai, 519000 People’s Republic of China; 3 Department of Ophthalmology, The Fifth Affiliated Hospital, Sun Yat-Sen University, No. 52 Mei Hua Dong Road, Zhuhai, 519000 People’s Republic of China; 4 Department of Interventional Radiology and Vascular Anomalies, Guangzhou Women and Children’s Medical Center of Guangzhou Medical University, Guangzhou, 510627 People’s Republic of China; 5 Department of Interventional Radiology, Guangdong Provincial People’s Hospital, Guangdong Academy of Medical Sciences, Guangzhou, 510000 People’s Republic of China; 6 United Laboratory of Fifth Affiliated Hospital and BGI, The Fifth Affiliated Hospital of Sun Yat-Sen University, Zhuhai, 519000, People’s Republic of China

**Keywords:** RB1, Mutation, Retinoblastoma, Genetics

## Abstract

**Purpose:**

To identify the spectrum of *RB1* gene mutations in 114 Chinese patients with retinoblastoma.

**Methods:**

Genomic DNA was extracted from the peripheral blood of 114 Rb patients. Polymerase chain reactions (PCRs) followed by direct Sanger sequencing were used to screen for mutations in the *RB1* gene, which contains 26 exons with flanking intronic sequences, except exon 15. Clinical data, including gender, age at diagnosis, laterality of ocular lesions, and associated symptoms, were recorded and compared.

**Results:**

We identified five novel mutations in the *RB1* gene. Twenty-five other mutations found in this study have been previously reported. A higher rate of *RB1* mutations, with 47.3% of mutations among bilaterally affected patients vs. 6.8% within unilaterally affected patients, was also observed (*p* < *0.0001*). Bilaterally affected patients were diagnosed earlier when compared to unilaterally affected patients (11 ± 7 months versus 20 ± 14 months, *p* = *0.0002*). Furthermore, nonsense mutations were abundant (*n* = 14), followed by frameshift mutations (*n* = 8), splicing site mutations (*n* = 5), while missense mutations were few (*n* = 3).

**Conclusions:**

We found five novel mutations in *RB1* genes, which expands the mutational spectrum of the gene. Children with bilateral Rb exhibited higher mutation rates and were diagnosed earlier than those with unilateral Rb. These findings will inform clinical diagnosis and genetic therapeutic targeting in Rb patients.

**Supplementary Information:**

The online version contains supplementary material available at 10.1007/s10792-022-02341-2.

## Introduction

Retinoblastoma (Rb; OMIM 180200), the most common primary intraocular malignancy in children, has a worldwide incidence rate of 1:15,000—20,000 live births [1]. About 95% of Rb cases are diagnosed before the age of five [2]. In developed countries, due to effective modern treatment, this disease is associated with a lower mortality rate and improved outcomes. In contrast, in developing countries, due to lack of prompt diagnosis and effective treatment, Rb patients have a 40–70% mortality rate and an increased risk of enucleation [3]. In addition, Rb survivors have higher risks of secondary tumors [4], including bone and soft tissue sarcomas, melanomas, leukemia, lung cancer, uterine leiomyosarcoma, and radiotherapy-related central nervous system tumors [5]. Secondary tumors adversely affect the quality of life and survival rate of Rb patients.

Mutations in *RB1*, the first described tumor suppressor gene located on chromosome 13q14.2, have been associated with heritable Rb [6, 7]. Mutated *RB1* gene may result in a loss of function of the retinoblastoma protein (pRB). Moreover, pRB knockdown was shown to promote tumor cell proliferation and repressed E2F activities [8]. Based on Knudson’s two-hit hypothesis, Rb development is mainly caused by mutations of both *RB1* alleles [9]. Individuals with *RB1* germline mutations are genetically predisposed to Rb [3]. Moreover, the types and frequencies of mutated *RB1* in children developing Rb vary geographically. Therefore, identification of *RB1* (Gene ID:5925; NM_000321) is key in genetic Rb testing and screening [10]. Genetic testing of the *RB1* gene is inevitable for early diagnosis in heritable Rb to provide timely therapies and improve prognosis [11]. However, in China, limited data are available on *RB1* mutations for prompt screening of all *RB1* mutations, avoiding false negatives in genetic testing of Rb patients, and informing the provision of effective therapies.

In this study, we characterized the spectrum of *RB1* mutations in children with Rb in China. Furthermore, the data generated in this study will form the basis for effective genetic counseling of Rb patients and their families, thereby improving Rb diagnosis and prognosis.

## Material and methods

### Patients

This study included 114 patients with retinoblastoma from Southern China. Clinical diagnosis of retinoblastoma was based on the clinical features and confirmed by an expert pathologist. All patients with Rb were diagnosed and treated according to the latest China guidelines for the diagnosis and treatment of retinoblastoma. Clinical data of all patients were collected from the hospital information system, including age, gender, tumor laterality, age at diagnosis, and associated symptoms (Table 1). All participants gave their written informed consent. The study was approved by the Ethics Committee of The Fifth Affiliated Hospital, Sun Yat-sen University, and was conducted in accordance with the principles of the 1975 Declaration of Helsinki.

**Table 1:** Clinical characteristics of the Rb patients

Characteristics	Total(*n* = 114)	Unilateral (*n* = 59)	Bilateral (*n* = 55)	*P values*
*Gender*				0.0859
Male	68 (59.6%)	40 (67.8%)	28 (50.9%)	
Female	46 (40.4%)	19 (32.2%)	27 (49.1%)	
*Side of Rb*				
Right eye	29 (25.4%)	29 (49.2%)	–	–
Left eye	30 (26.3%)	30 (50.8%)	–	–
Both	55 (48.3%)	–	55 (100%)	–
*Age at Diagnosis (months)*				
Median age	15 ± 12	20 ± 14	11 ± 7	0.0002
< = 24	91 (79.8%)	38 (64.4%)	53 (96.4%)	–
> 24	23 (20.2%)	21 (35.6%)	2 (3.4%)	–
*Germline mutation*				< 0.0001
Mutation	30 (26.3%)	4 (6.8%)	26 (47.3%)	–
No mutation	84 (73.7%)	55 (93.2%)	29 (52.7%)	–

### Mutation detection

#### PCR

Genomic DNA was extracted from blood samples of patients firstly diagnosed with Rb using the iPure blood genomic DNA kit (IGEbio, CZ313-S). The extracted DNA was quantified using Nanodrop 2000 (Nanodrop, Wilmington, DE, USA). The primers used for detecting *RB1* mutations (shown in Supplementary Table S1) were designed using Primer Premier 3.0 software for 26 translating exons and the adjacent intron–exon regions of the *RB1* gene, except exon 15. The PCR had a total volume of 20 µl, including 10 µl HiFipfu polymerase (IGEbio, P300-S), 7 µl nuclease-free water, 1 µl forward and reverse primers (10 uM) each, and 2 µl DNA template. A BIO-RAD T100 TM Thermal cycler (Bio-Rad, Gladesville, Australia) and a touchdown PCR protocol were used for amplification. The PCR conditions were as follows: initial denaturation at 94 °C for 5 min followed by 10 cycles of denaturation at 94 °C for 30 s, annealing at 65 °C for 30 s, with the temperature of the heating block decreased by 1 °C per cycle, and then extension at 72 °C for 40 s. This was then followed by 25 cycles of denaturation at 94 °C for 30 s, annealing at 55 °C for 30 s, extension at 72 °C for 40 s and final extension at 72 °C for 3 min.

#### Sanger sequencing and sequence analysis

The PCR products were visualized by 1.5% agarose gel electrophoresis. The target band was excised, after which the DNA was purified and sequenced with ABI3730XL (Applied Biosystems, Foster City, CA, USA) using the BigDye method. Nucleotide sequences of the *RB1* gene (GenBank L11910.1) were used as the reference. A Sequence Scanner, version 1.0 (Applied Biosystems, Streetsville, Ontario, Canada), and chromas (2.6.5) were used to align each exon’s sequence with the reference sequence to annotate mutations. Variants were described based on the latest nomenclature for the description of sequence variants (Human Genome Variation database: http://www.HGVS.org/varnomen). Additional information on *RB1* gene mutations was obtained from Clinvar (https://www.ncbi.nlm.nih.gov/clinvar/), Human Gene Mutation Database (HGMD: http://www.hgmd.cf.ac.uk/ac/), LOVD (http://www.lovd.nl/3.0/home), gnomAD (http://gnomad.broadinstitute.org/), ensemble, and dbSNP. pRB protein structures were retrieved from UniProt (http://www.uniprot.org/uniprot, P06400). Open reading frame (ORF) and frameshift mutation predictions were performed using the NCBI ORF finder (https://www.ncbi.nlm.nih.gov/orffinder). The pathogenicity of variants was interpreted according to ACMG guidelines.

### Statistical analysis

Student’s *t* test was performed to compare differences between continuous variables. Categorical variables were compared using Chi-square or Fisher’s exact tests. *p* ≤ 0.05 was considered statistically significant. Data analysis was performed using the SPSS software, version 25.0.

## Results

### Clinical characteristics

A total of 114 Chinese patients with Rb were recruited for this study, including 68 males and 46 females (59.6% vs 40.4%). A summary of the cases and their clinical characteristics are shown in Table 1. Fifty-five patients were bilaterally affected, while fifty-nine patients were unilaterally affected (48.2% versus 51.8%). In detail, unilateral cases were observed in 40 males and 19 females, while bilateral cases were observed in 28 males and 27 females (*p* = *0.0859*). In unilateral cases, distributions of left and right ocular lesions were relatively similar (30 versus 29, respectively). The age at diagnosis ranged from 1 to 64 months old, with an average age of 14 months. Moreover, 91 patients (79.8%) had Rb onset before the age of 2 years, while 23 patients (20.2%) had Rb onset after 2 years of age. Most of the bilaterally affected patients (53/55, 96.4%) were diagnosed before 2 years. Mean age at diagnosis for patients with bilateral Rb was lower than for unilateral cases (11 ± 7 months versus 20 ± 14, *p* < *0.0001*). This finding is consistent with those of previous studies [3, 12, 13]. Leukocoria was the most common initial symptom in Rb cases, accounting for 65.8% of all cases, followed by squint (21.9%). Several patients presented with other symptoms, including redness, accompanied by tearing, vision diminution, calcification, and white spots.

### Mutations

#### Novel mutations

**Fig. 1:**
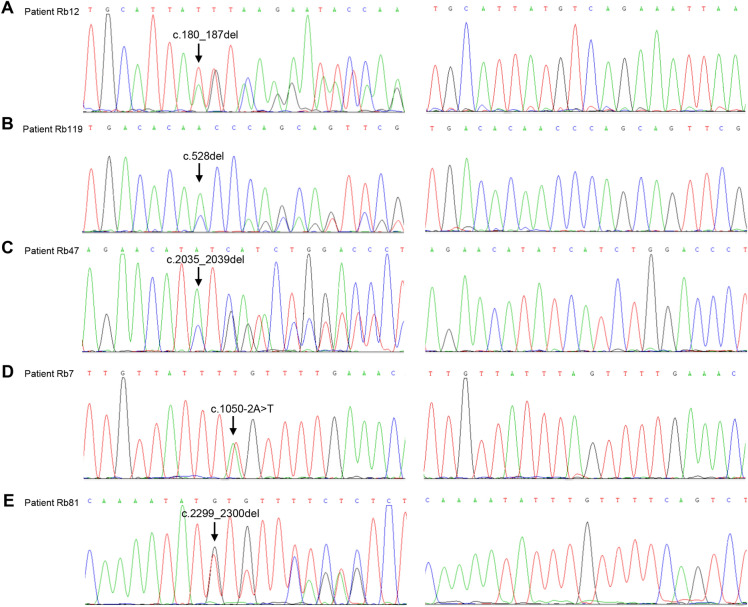
Sequencing chromatograms illustrating novel mutations in the patients. The left panels show the mutations identified in Rb patients, while the right panels show the wildtype *RB1* sequences. Arrow indicates the mutated site. **A** c.180_187del heterozygous mutation in patient Rb12. **B** c.528del heterozygous mutation in patient Rb119. **C** c.2035_2039del heterozygous mutation in patient Rb47. **D** c.1050-2A > T heterozygous mutation in patient Rb7. **E** c.2299_2300del heterozygous mutation in patient Rb81

**Fig. 2:**
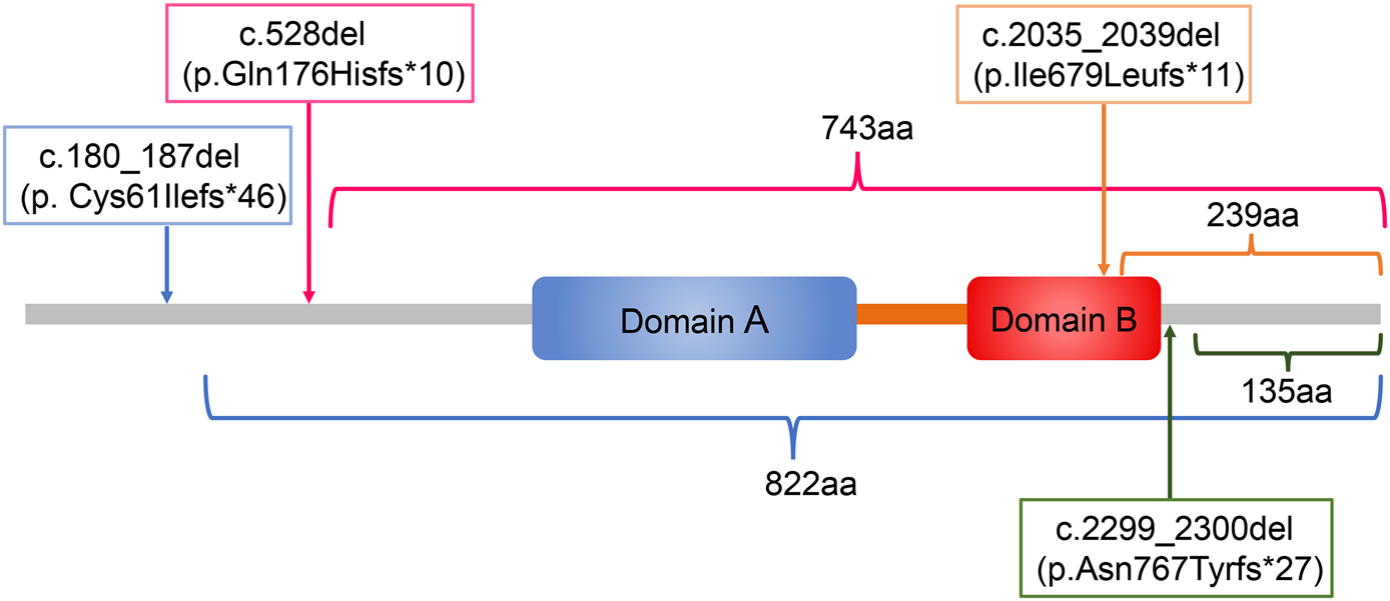
Effects of the four novel frameshift mutations on the RB1 protein. c.180_187del (p.Cys61Ilefs*46) mutations caused a frameshift change and a premature stop codon at amino acid 106, truncating 822 amino acids at the C terminus of the RB1 protein. c.528del (p.Gln176Hisfs*10) mutations caused a frameshift change and a premature stop codon at amino acid 185, truncating 743 amino acids at the C terminus of the RB1 protein. c.2035_2039del (p.Ile679Leufs*11) mutations caused a frameshift change and a premature stop codon at amino acid 689, truncating 239 amino acids at the C terminus of the RB1 protein. c.2299_2300del (p.Asn767Tyrfs*27) mutations caused a frameshift change and a premature stop codon at amino acid 793, truncating 135 amino acids at the C terminus of the RB1 protein

A total of five novel mutations were identified in our Rb cases. Sequencing analysis of the five novel variants is presented in Fig. 1. There were four frameshift mutations and one splice site mutation consisting of c.180_187del, c.528del, c.2035_2039del, c.2299_2300del, and c.1050-2A > T. All mutations were identified in bilaterally affected Rb patients with a mean age at diagnosis of 13.6 months. The splice site mutation, c.1050-2A > T, presenting at 3’ splice site of intron 10, altered the sequence from AG to TG and disrupted the canonical splice site. Intriguingly, the four novel frameshift mutations were predicted to result in a truncated RB protein arising from a premature stop codon in the reading frame and the loss of coding exons. Furthermore, three of the four frameshift mutations resulted in premature termination of amino acid translation in the conserved pRB pocket region, encoding transcriptional regulation and forming the repressor motif [14]. As shown in Fig. 2, c.180_187del (p.Cys61Ilefs*46) and c.528del (p.Gln176Hisfs*10) mutations occurred in exon2 and exon5, causing a frameshift variant and a premature stop codon at amino acid 106 and 185, and truncating 822 and 743 amino acids, respectively, at the C terminus of the RB1 protein. In addition, c.2035_2039del (p.Ile679Leufs*11) was located at domain B of the pRB pocket, resulting in premature translational termination at position 689, subsequently truncating 239 amino acids of the predicted protein. The other novel frameshift mutation, c.2299_2300del (p.Asn767Tyrfs*27), generated a frameshift and premature termination of the open reading frame.

### Other mutations

Twenty-five other previously reported *RB1* mutations we found in this study are shown in Table 2. The most common mutational type was nonsense mutations (fourteen of twenty-five; 56.0%), followed by frameshift (three of twenty-five; 12.0%), splice mutations (three of twenty-five; 12.0%), and missense mutation (three of twenty-five, 12.0%).

**Table 2:** A summary of *RB1* mutations in the 30 Rb patients

Patient ID	Location	Mutational type	Mutation	Change in protein	Laterality	Status
Rb95	Exon1	Frameshit	c.19dup	p. Arg7Profs*24	Bilateral	Reported
Rb43	Exon1	Missense	c.137G > A	p.Arg46Lys	Bilateral	Reported
Rb12	Exon2	Frameshit	c.180_187del	p. Cys61Ilefs*46	Bilateral	Novel
Rb25	Exon3	Nonsense	c.334G > T	p.Glu112*	Bilateral	Reported
Rb82	Exon4	Nonsense	c.446C > G	p.Ser149*	Bilateral	Reported
Rb119	Exon5	Frameshit	c.528del	p.Gln176Hisfs*10	Bilateral	Novel
Rb110	Exon8	Nonsense	c.751C > T	p.Arg251*	Bilateral	Reported
Rb103	Exon8	Nonsense	c.763C > T	p.Arg255*	Bilateral	Reported
Rb107	Exon8	Nonsense	c.763C > T	p.Arg255*	Unilateral	Reported
Rb84	Exon10	Nonsense	c.958C > T	p.Arg320*	Bilateral	Reported
Rb7	Intron10	Splicing	c.1050-2A > T	–	Bilateral	Novel
Rb16	Intron12	Splicing	c.1215 + 1G > A	–	Bilateral	Reported
Rb2	Exon13	Frameshit	c.1236del	p.Glu413Lysfs*4	Unilateral	Reported
Rb117	Exon13	Missense	c.1332G > C	p.Gln444His	Bilateral	Reported
Rb89	Exon14	Nonsense	c.1333C > T	p.Arg445*	Bilateral	Reported
Rb36	Exon14	Nonsense	c.1388C > G	p.Ser463*	Bilateral	Reported
Rb54	Intron16	Splicing	c.1498 + 1G > A	–	Bilateral	Reported
Rb92	Exon17	Frameshit	c.1585del	p.Tyr529Thrfs*3	Bilateral	Reported
Rb88	Exon17	Nonsense	c.1633G > T	p.Glu545*	Bilateral	Reported
Rb13	Exon18	Nonsense	c.1735C > T	p.Arg579*	Bilateral	Reported
Rb99	Exon18	Nonsense	c.1735C > T	p.Arg579*	Bilateral	Reported
Rb115	Exon18	Nonsense	c.1735C > T	p.Arg579*	Bilateral	Reported
Rb40	Exon19	Frameshit	c.1959dup	p.Val654Serfs*14	Bilateral	Reported
Rb24	Exon20	Missense	c.1981C > T	p.Arg661Trp	Bilateral	Reported
Rb47	Exon20	Frameshit	c.2035_2039del	p.Ile679Leufs*11	Bilateral	Novel
Rb37	Exon20	Nonsense	c.2042G > A	p.Trp681*	Unilateral	Reported
Rb17	Intron21	Splicing	c.2211 + 1 G > A	–	Bilateral	Reported
Rb126	Intron21	Splicing	c.2211 + 5 G > T	–	Bilateral	Reported
Rb70	Exon22	Nonsense	c.2242G > T	p.Glu748*	Unilateral	Reported
Rb81	Exon22	Frameshit	c.2299_2300del	p.Asn767Tyrfs*27	Bilateral	Novel
*Patients*						
Rb70's mother	Exon22	Nonsense	c.2242G > T	p.Glu748*	Normal	Reported

As shown in Table 3, in a total of 30 variants, the most commonly observed *RB1* gene alteration was nonsense mutation, accounting for 46.7% of the detected mutations, followed by frameshift mutations (26.6%), splicing alterations (16.7%), and missense mutations (10.0%). Mutations located at exons 8, 18, 20 exhibited the highest mutation frequencies and were, respectively, found in 3 patients. Mutations at exons 1, 13, 14, 17, 22, and intron 21 were, respectively, found in 2 patients. There were no mutations in exons 6, 7, 9, 11, 16, 22, and 24–27. Furthermore, 50.0% (15/30) of the detected mutations were located at regions encoding two conserved domains, A (residue 373–573) and B (residue 646–765), of the pRB pocket containing five nonsense, four splicing, four frameshift, and two missense variants. Specifically, 4/5 splice site mutations and 2/3 missense mutations were located at the pRB pocket region. The mutation rate in domain A located at exons 12–18 was 26.7% (8/30), while the mutation rate in domain B located at exons 19–23 was 23.3% (7/30). In addition, 21/30 mutations caused a premature stop codon on RNA transcripts resulting in a truncated protein.

**Table 3:** Distributions of mutations in *RB1* by type

Mutation type	Unilateral	Bilateral	Total
Nonsense	3(10.0%)	11(36.7%)	14/30(46.7%)
Frameshift	1(3.3%)	7(23.3%)	8/30(26.6%)
Splicing	0(0.0%)	5(16.7%)	5/30(16.7%)
Missense	0(0.0%)	3(10.0%)	3/30(10.0%)

Of the 14 cases with nonsense mutations, nine (64.3%) patients had C to T transitions in CGA codons in exons 8, 10, 14, 18, with several of these mutations affecting more than two unrelated patients. For instance, the c.1735C > T (p.Arg579*) mutation in exon 18 was detected in three unrelated patients, while the c.763C > T (p.Arg255*) mutation in exon8 was detected in two unrelated patients. Additionally, c.2242G > T(p.Glu748*) was detected in the proband’s mother, who did not suffer from Rb, indicating that this mutation was maternally inherited. Four of the eight reported frameshift mutations were novel mutations that had never been previously reported or characterized in mutated *RB1*. Moreover, four of the five splicing mutations occurred by substitution of G to A/T. Cases with splice site mutation were diagnosed at a younger age, ranging from 1 to 8 months.

### Genotype–phenotype correlations

**Fig. 3:**
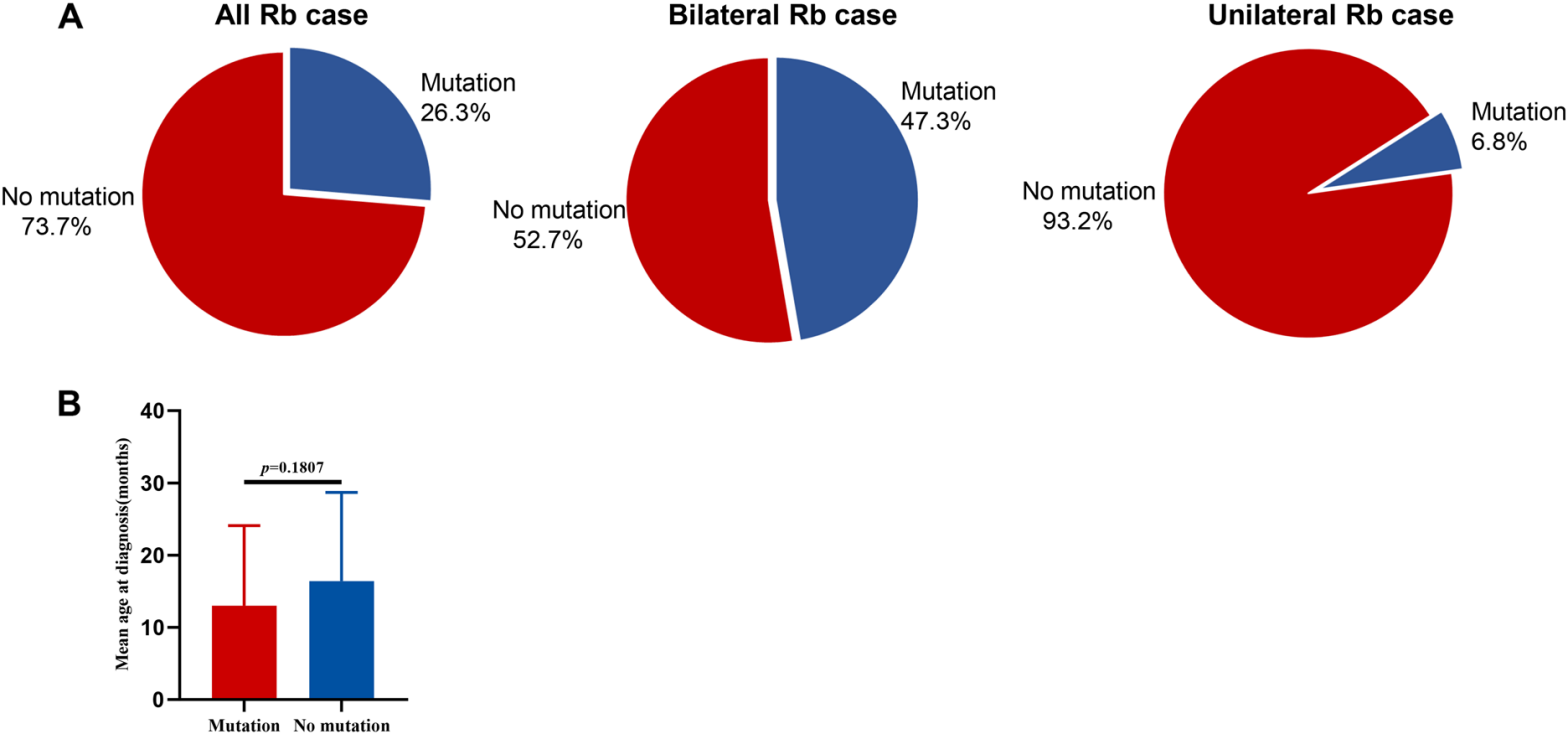
Mutation detection rate and mean age at diagnosis in retinoblastoma patients. **A** The left pie chart shows an overall mutation detection rate of 26.3% (30/114) in all Rb cases. The middle pie chart shows the mutation rate of 47.3% (26/30) in bilateral Rb cases. The right panel shows a mutation detection rate of 6.8% (4/59) in unilateral Rb cases. **B** Age at diagnosis of probands with positive (*n* = 30) or negative (*n* = 84) *RB1* mutation test results

Sequencing analysis was performed for the 26 exons and adjacent intronic regions, except exon 15. DNA analysis revealed 27 different causative *RB1* mutations in 30 patients (26.3%), which were identified in 4/59 (6.8%) patients with unilateral Rb and 26/55 (47.3%) patients with bilateral Rb (*p* < *0.0001*) (Fig. 3A). The mutation rate in the bilaterally affected patients was significantly higher than in the unilaterally affected patients, consistent with previous findings [15]. Mean age at diagnosis for patients with mutated *RB1* was 13 ± 11 months and 16 ± 12 months for patients without mutated *RB1*. Differences in age at diagnosis between the two groups were not significant (*p* = *0.1807*) (Fig. 3B).

## Discussion

In this study, we identified *RB1* gene mutations in 26.3% of the 114 recruited Chinese Rb patients. This included five novel mutations that had never been previously reported. Bilaterally affected Rb patients exhibited higher mutation rates and an earlier age at diagnosis than unilaterally affected patients.

Most frameshift or nonsense mutations in inherited diseases or cancers result in premature termination codons (PTCs) [16]. In this study, nonsense and frameshift mutations were the major mechanisms of *RB1* inactivation, accounting for 73.3% of all *RB1* mutations. This finding is consistent with previously reported rates of 50–80.32% [4, 12, 17–19]. Furthermore, 50.0% of all *RB1* mutations lie in the A/B “pocket” domain of pRB, which can bind E2F transcription factors and promote protein–protein interactions [20]. This mutation rate was comparable to previously reported rates of 40–78.95% [4, 12, 17, 21, 22]. In addition, subtle mutations may result in partial inactivation of pRB, decreasing protein stability. Therefore, variants occurring in or adjacent to known conserved protein domains have a great influence on disease onset [23].

Premature stop codons resulting in loss-of-function (LOF) alleles are strongly associated with inactivation of the RB protein [24]. Accordingly, frameshift mutations with premature translation termination might contribute to the onset of diseases by degrading *RB1* transcripts, resulting in the complete loss of pRb. In this study, four of the five novel *RB1* variants were frameshift mutations and were reported in patients with bilateral lesions. According to the ACMG standards and guidelines [25], all novel *RB1* frameshift variants were pathogenic. This finding supports the postulate that frameshift mutations are highly associated with retinoblastoma disease. Studies should evaluate the specific impacts of these novel mutations on protein functions.

A marginally lower degree of *RB1* germline mutation rate was reported in this study, compared to studies done in other countries [17–19, 26, 27]. Our findings are consistent with several studies conducted in China, with 21–27.1% of total *RB1* mutation rates detected using Sanger sequencing alone [7, 28, 29]. Therefore, we hypothesized that geographical variations or race accounted for these differences. The heterogeneity in mutation detection rates can also be explained by the different techniques, such as MLPA and next-generation sequencing [30], used to detect mutations. In addition, the fact that we missed sequencing analysis of the promoter region and exon 15 due to the poly (A) and poly (T) sequence on both sides could also explain this difference.

Retinoblastoma is a highly complex disease. Single-nucleotide variants, large rearrangements, and promoter hypermethylation of the *RB1* gene have a role in its development [31, 32]. Furthermore, low-level mosaic and deep intronic variants have been identified [33]. Besides, other genes may also be attributed to the Rb phenotype. For instance, *MED4*, a synthetic lethal target in tumors, explains the low penetrance of retinoblastoma [34]. In addition, amplification of the *MYCN* oncogene contributes to retinoblastoma [35]. Besides, retinoblastoma has also been associated with *MDM2*, the first modifier gene so far identified in retinoblastoma [36]. Further, the *PIN1* gene that alters pRB phosphorylation is also important for Rb development [37]. Despite extensive research, the exact pathomechanisms of Rb remain elusive and should be investigated further. Given the relatively high negative rate of *RB1* mutations in this study, we will perform whole-exome sequencing (WES) in samples obtained from patients without *RB1* mutations to explore other possible disease-causing genes and variants. We postulate that our findings will elucidate the pathogenesis of Rb.

The spectrum of *RB1* mutations in this study will facilitate the development of better-targeted therapies and inform on optimal disease management as well as effective treatment of Rb patients [38]. Prenatal diagnosis and family screening should be performed to identify individuals with a genetic predisposition to Rb [39].

## Supplementary Information

Below is the link to the electronic supplementary material.

Supplementary file1 (DOCX 27 KB)

## Data Availability

The findings of this study are included within the article and are available from the corresponding author upon reasonable request.

## Abbreviations

Rb: Retinoblastoma
PTCs: Premature termination codons
LOF: Loss of function
MLPA: Multiplex ligation-dependent probe amplification
WES: Whole-exome sequencing
