# Supplementary material for: Five novel RB1 gene mutations and genotype–phenotype correlations in Chinese children with retinoblastoma
Source: Int Ophthalmol. 2022 Aug 12;42(11):3421–30. doi: 10.1007/s10792-022-02341-2 (PMC9587959; doi:10.1007/s10792-022-02341-2)
Supplement: Supplementary file 1 — Supplementary file1 (DOCX 27 KB) [file 10792_2022_2341_MOESM1_ESM.docx]

**Table S1.** **PRIMERS FOR RT-PCR AND CDNA SEQUENCING.**

| Region | Primer | Sequence(5'-3') | Annealing(°C) | Amplicon（bp） | Notes |
| --- | --- | --- | --- | --- | --- |
| Exon1 | RB1-F1 | tttctcaggggacgttgaaa | 56.99 | 483 |  |
|  | RB1-R1 | tgtcaagttgaagccgagac | 58.13 |  |  |
| Exon2 | RB1-F2 | actgtgtggtatccttattttgg | 56.51 | 500 |  |
|  | RB1-R2 | tttaatgttagcagaggtaaatttcc | 56.27 |  |  |
|  | RB1-F2-2 | ttcacagaagtgttttgctgctt | 59.81 | 912 |  |
|  | RB1-R2-2 | gtttggtgggaggcatttatgg | 59.83 |  |  |
|  | RB1-F2ss | gaaacaagtatgtactg | 48.00 |  | sequencing primer |
| Exon3 | RB1-F3 | agaaggatgtgttacaaatatacagt | 56.45 | 400 |  |
|  | RB1-R3 | agttcactatttggtccaagttct | 58.06 |  |  |
| Exon4 | RB1-F4 | agtagtgatttgatgtagagctga | 57.22 | 376 |  |
|  | RB1-R4 | tgagctaacattaaaagggacaag | 57.18 |  |  |
| Exon5 | RB1-F5 | tgggaaaatctacttgaactttgt | 56.72 | 300 |  |
|  | RB1-R5 | agcctgctataatcgatcaaact | 57.78 |  |  |
| Exon6 | RB1-F6 | tttgcagcttctcatggtca | 57.72 | 695 |  |
|  | RB1-R6 | aatgccgattatccctgggt | 58.85 |  |  |
| Exon7 | RB1-F7 | accatgctgatagtgattgttga | 58.15 | 325 |  |
|  | RB1-R7 | tcctgtcagccttagaacca | 57.98 |  |  |
| Exon8 | RB1-F8 | ggagcagagtagaagagggatg | 59.37 | 570 |  |
|  | RB1-R8 | acttttcagtgattccagagtga | 57.70 |  |  |
| Exon9 | RB1-F9 | tctaacttaccctgcattgttca | 58.82 | 297 |  |
|  | RB1-R9 | gctagattcttcttgggcaaa | 58.59 |  |  |
| Exon10 | RB1-F10 | attgcatgcgaactcagtgt | 58.47 | 459 |  |
|  | RB1-R10 | ctgttataggacacacaattcaca | 56.96 |  |  |
| Exon11 | RB1-F11 | gcagctgggtcatctattttct | 58.45 | 363 |  |
|  | RB1-R11 | ccacacctggccttcaatatata | 57.76 |  |  |
| Exon12 | RB1-F12 | ccacagtcttatttgagggaatg | 57.18 | 481 |  |
|  | RB1-R12 | agttgcaaggaagaatggtga | 57.77 |  |  |
|  | RB1-F12-2 | acttgggagatggaaaacatttca | 58.85 | 591 |  |
|  | RB1-R12-2 | acctggaattgaagatcgagca | 59.76 |  |  |
| Exon13 | RB1-F13 | tgttcagtagttgtggttacct | 56.97 | 400 |  |
|  | RB1-R13 | ggcagcagggatatagtatctga | 58.97 |  |  |
| Exon14-16 | RB1-F14-15-16 | ctcttgagcccaggagtgtg | 61.00 | 976 |  |
|  | RB1-R14-15-16 | cccaagatggcctcaaataa | 59.89 |  |  |
|  | RD1-R14s | atggattccattactcgg | 55.00 |  | sequencing primer |
|  | RB1-F14s | gtttgtagcgatacaaac | 53.00 |  | sequencing primer |
|  | RD1-F16s | cctttagcaaacttctgaatg | 57.00 |  | sequencing primer |
| Exon17 | RB1-F17 | ggctatttcctatgagtccgt | 56.97 | 466 |  |
|  | RB1-R17 | ttccctatatgttcttgaggtaga | 56.11 |  |  |
|  | RD1-F17s | taggaagtacatctcag | 49.00 |  | sequencing primer |
| Exon18 | RB1-F18 | ctgacttttaaattgccactgtca | 58.17 | 498 |  |
|  | RB1-R18 | aaactgccaactctgccatg | 59.04 |  |  |
| Exon19 | RB1-F19 | ccagcttgcatttaaatagtctgc | 58.96 | 380 |  |
|  | RB1-R19 | tgcccagtaatgtacctagaaagt | 59.22 |  |  |
| Exon20 | RB1-F20 | ttgaggctaaaagaaagaaaatgg | 60.12 | 323 |  |
|  | RB1-R20 | tgtgaatttacataataaggtcagaca | 58.23 |  |  |
| Exon21 | RB1-F21 | ttgaggctaaaagaaagaaaatgg | 56.10 | 323 |  |
|  | RB1-R21 | tgtgaatttacataataaggtcagaca | 57.05 |  |  |
| Exon22-23 | RB1-F22-23 | ctgcctggctatttctctcaatc | 59.19 | 807 |  |
|  | RB1-R22-23 | atcttgcgttgcttaagtcgtaa | 59.01 |  |  |
|  | RB1-23Fs | tgcttccaccagggtaggtc | 65.00 |  | sequencing primer |
| Exon24 | RB1-F24 | tggtattcctaatagttcagaatgatg | 58.98 | 250 |  |
|  | RB1-R24 | gcaatatgcctggatgaggt | 59.92 |  |  |
| Exon25-26 | RB1-F25-26 | tgcctgatttttgacacacc | 59.55 | 910 |  |
|  | RB1-R25-26 | acaaacctgccaactgaagaa | 59.77 |  |  |
| Exon27 | RB1-exon27cds-F | aagaccactgcttttgcaaggt | 61.21 | 369 |  |
|  | RB1-exon27cds-R | agtggcttaggaatcacccaaac | 60.81 |  |  |
|  | RB1-F27-1 | cagccacttgccaacttacc | 59.40 | 1288 |  |
|  | RB1-R27-1 | ggctttgaacatgccagtg | 57.50 |  |  |
|  | RB1-F27-2 | tgtttctgggtcctgaagaat | 56.83 | 560 |  |
|  | RB1-R27-2 | agagccccttaaaggctagg | 58.49 |  |  |
